# The quality of service provision to newborns in the primary healthcare, West Gojjam zone, North-West of Ethiopia: a cross-sectional survey

**DOI:** 10.1186/s12887-022-03272-8

**Published:** 2022-04-13

**Authors:** Bizuhan Gelaw Birhanu, Johanna Mmabojalwa Mathibe-Neke

**Affiliations:** 1 Community Newborn and Child Health Specialist, UNICEF Ethiopia Country Office, Addis Ababa, Ethiopia; 2 Department of Health Studies, College of Human Sciences, University of South Africa (UNISA), Pretoria, South Africa

**Keywords:** Quality of service, Factors, Neonate/newborn, Primary healthcare units, West Gojjam, Ethiopia

## Abstract

**Background:**

During 2019, neonatal conditions in Ethiopia accounted for 55% of under-5 deaths, with 33 neonatal deaths occurring for every 1000 live births. More than 80% of all newborns deaths are caused by preventable and treatable conditions with available interventions. In Ethiopia, mortality rates for newborn babies have remained stubbornly high over the decades.

**Objective:**

This research aimed to assess the quality-of-service provision for newborns in the primary healthcare units in the North-West of Ethiopia.

**Methods:**

A cross-sectional survey design was employed. Interviewer-administered questionnaires were administered to 221 health workers and health extension workers, and health facility readiness assessment was done in 142 health facilities including 3 Primary hospitals, 76 Health centres and 63 Health posts from April to July 2017. Data was entered into the EpiData 3.1, exported to SPSS and STATA for analysis. Descriptive and regression multivariate analysis was applied.

**Results:**

Out of the 10 quality of newborn care variables, 8.7 [95%CI: 6.03–11.303], the highest mean was achieved by primary hospitals, followed by urban health centres with a 6.4 mean [95%CI:5.168–7.601]. However, nearly half of the rural health centres were providing quality of newborn care at the mean of 5.7 [95%CI: 5.152–6.18], and below half was provided by health posts, 4.5 [95%CI: 3.867–5.116]. From the seven emergency newborn care signal functions, primary hospitals had a higher mean score, 6.3 [95%CI: 6.007–7.325] and rural health centres had the lowest mean score, 2.3 [95%CI: 2.043–2.623]. The availability of essential equipment is also significantly associated with the quality of neonatal care provision in the health facilities (*p* < 0.05). Overall, the effectiveness of the neonatal healthcare services has a significant association with the health facility readiness score [95%CI: 0.134–0.768].

**Conclusion:**

The quality of newborn care was high at the higher-level health facilities and lower in the lower-level health facilities such as rural health centres and health posts, where these facilities are an entry point to the health system and are expected to provide the essential newborn care services to the majority of the rural communities. In addition, the provision of emergency newborn care signal functions was critically low in rural health centres where these are referral receiving health facilities from health posts. Thus, rural health centres and health posts should be targeted to improve their readiness to provide the quality of services for newborns as per their expected level of care.

## Background

The first 28 days of life – the neonatal period – is the most vulnerable time for children under the age 5. At the global level, under-5 years of children face the highest risk of dying in their first month of life at,17 deaths per 1000 live births (LBs) in 2019 and the neonatal deaths accounted for 47% of global under-five deaths. About a third of all neonatal deaths occurring within the first day after birth, and close to three-quarters occurring within the first week of life [1]. In 2019, neonatal conditions in Ethiopia account for 55% of under-5 deaths, with 33 neonatal deaths occur for every 1000 LBs. Mortality rates for newborn babies have remained stubbornly high and about 109,000 newborns die every year in Ethiopia [1, 2]. More than 80% of all newborn deaths are caused by preventable and treatable conditions with available interventions [3, 4]. In Ethiopia, in 2017, the most common causes of newborn death are intrapartum related events (birth asphyxia & birth trauma) (30%), preterm birth complications (26%), sepsis & tetanus (18%), pneumonia (8%), diarrhoea (1%), congenital abnormalities (11%) and other conditions (7%) [5].

In addition, quality of care is not offered for newborns and mothers who are already visiting the health system. Too many mothers and newborns miss out on key interventions that can save their lives [6]. The assessment done on newborn care in four countries [7] reported that within primary healthcare and referral level health facilities, the newborn care services provision is found to be in poor quality; and this poor quality is exacerbated by deficient competency by health professionals. In the first level healthcare service provision and referral level, the quality of newborn care is generally substandard; and the limited knowledge and skills among providers contributed to poor quality of newborn care [7].

In Ethiopia, in 2019, 48% of births occurred in the health facilities and only 35% of newborns received postnatal care (PNC) check-up within 48 h after birth [2]. In this country, there is a wide regional difference in neonatal mortality, where neonatal mortality rates range from a low level of 21 per 1000 live births in Addis Ababa (the capital city of Ethiopia) to a high rate of 54 per 1000 live births in the Amhara region. Before reaching the age of 1 month, 17% more newborn infants die in the Amhara region than in the nation [8]. To fast-track the reduction of deaths in under-5 years, prioritizing newborn conditions is very crucial for Ethiopia.

## Objective

This research aimed to assess the quality of service provision for newborns in the primary healthcare units in the North-West of Ethiopia.

## Methods

### Study design and setting

A cross-sectional survey design [9, 10] was employed to assess the quality of newborn care in the primary health care units (PHCUs) of West Gojjam zone, North-West of Ethiopia. West Gojjam is one of the 11 administration zones in the Amhara regional state, with 2,463,004 estimated total population for 2015, consisting of fifteen districts (*woredas*). In 2017, the zone has three primary hospitals (PHs), 91 health centres (HCs) and 373 health posts (HPs). Since there were only three primary hospitals, all were included in the study. The Ethiopian health care delivery system is organised into three tier systems. At the bottom of the health system, the primary health care delivery encompasses health posts, health centres and primary hospitals. The health post provides service to a population of 3000 to 5000, health centre that provides service to an estimated population of 15,000 to 25,000, and primary hospital also provides services to about 100,000 catchment population [11, 12]. Maternity care (antenatal care, delivery, and postnatal care), and newborn care services through the newborn corner, integrated management of newborn and childhood illnesses (IMNCI), and essential newborn care are key components of services provided at the health centres and primary hospitals. In addition, newborn intestine care unit level one and emergency surgery (including Caesarean sections and blood transfusions) are available at the primary hospitals. In addition, antenatal care, postnatal care, integrated community case management (iCCM) of common childhood illnesses and community based newborn sepsis management are the responsibility of Health Extension Workers at community level in the health post or Kebele (the lowest administrative unit, with 5000 population) [11–13].

### Study population

The targeted population was all health workers in PHs and HCs who were working in maternity ward (delivery and early post-natal) and under-five clinics and all health extension workers (HEWs) who were working in the HPs in selected heath facilities in all the fifteen *woredas* (Districts) of the administrative zone.

### Inclusion and exclusion criteria

All functional PHCUs and all health workers (HWs) and HEWs working maternity & early postnatal care ward and under-five clinic/outpatient department were included in the selected *woredas*. However, PHCUs which were not functional for various reasons and professionals who didn’t work in maternal and newborn health services were excluded.

### Sample size determination and sampling procedure

**Fig. 1:**
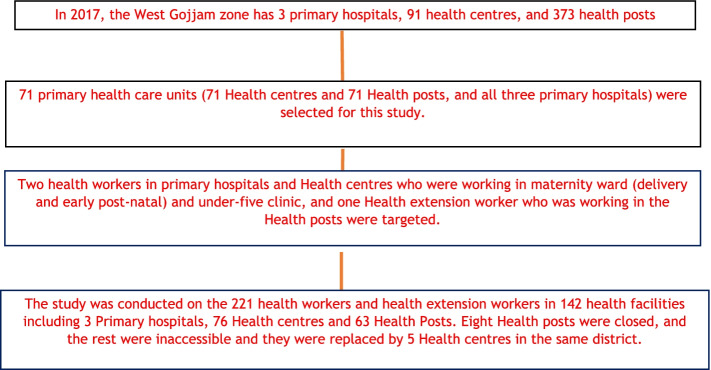
Participants follow chart

StatCalcEpi info version 7 statistical software was used to calculate the sample size. The PHCUs/HCs were the study units and the following assumptions were considered to calculate the sample size: ninety-one PHCUs as a total population size; prevalence is considered as 50% the reason that no similar study was done so far; confidence limits is 5%; confidence level is 95%; design effect is 1 and clusters is considered as 1 to obtain the final sample of 71 PHCUs (71 HCs and 71 HPs). Having all the list of PHCUs from the *zone* and confirmed *by* the respective *woredas*, the PHCUs were selected by a simple random sampling technique. In addition, one HP was selected by same methods with the available HPs from each selected PHCU catchment’s. From the selected PHCU, two health workers in PH and HC who were working in maternity ward (delivery and early post-natal) and under-five clinic and one HEW who was working in the HP were selected (Fig. 1).

### Data collection tool

The questionnaire was composed of three major parts, including background and health facility identification, maternity unit (antenatal care, delivery, and postnatal care and neonatal health services and management of sick neonates in under-five clinics. Overall, this questionnaire was used to solicit information from a sample of health service providers on their qualification, position, training, experience and supervision they received, and a set of service specific knowledge questions, case scenarios to capture correct practices on resuscitation, immediate newborn care of stabilized baby, thermal care, immediate care of very low birth weight and breastfeeding advice were included. In addition, to collecting information on general facility readiness, focusing on maternal and newborn health such as facility infrastructure (electricity, water), staffing, availability of basic supplies and equipment, medicines and commodities, infection prevention, and laboratory diagnostic under- different subtitles. The questioner was designed for both interview and observation to confirm the availability of the requested items.

### Data collection

From April to June 2017, questionnaires were administered by the trained data collectors at 221 HWs and HEWs in 142 health facilities including 3 PHs, 76 HCs and 63 HPs; two health workers per PH and HC and one HEW per HP. All the 15 *woredas* in the West Gojjam zone were represented by the selected health facilities. The questionnaire was composed of questions that require observation and verification by checking the availability and functionality of essential maternal and newborn supplies, equipment, medicines, and documents in the health facility. There were also questions that were checking the knowledge and practice of the healthcare providers that inquired, avoiding probing/seeing from the list of options. As a result, the researchers employed an interviewer-administered questionnaire with a combination of interviews of the health care providers and observation & verification for the health facility assessment.

### Clinical scenario to assess the quality of essential newborn care

Among the health workers (HWs) who were working in the maternity ward in the primary hospitals and health centres, and health posts who might imminent labour, a clinical case scenario was offered to understand the quality of newborn resuscitation when they are facing a similar scenario in their respective health facilities. HWs were asked about the type of services they perform if they are facing a woman in labour at their facility with the foetal heart rate being more than 160 beat per minute, on examination, her cervix is fully dilated, and the baby’s head is on the perineum. Preparing the mother for immediate delivery and resuscitating the baby was spontaneously mentioned by 100 (80.6%) and 62 (50%) of HWs respectively. The scenario was continued as if the baby is delivered and with normal weight, but baby does not cry after delivery. The HWs were asked about the services provided if the baby is not crying.

From the above clinical scenario, 13 actions from the three domains including actions for resuscitation, follow-up care after resuscitation and thermal care were expected as a spontaneous response by the respondents. Each main domain had 5, 3 and 5 actions respectively. Each main category had a total score of 10; and all the three domains had a total sum score of 30. Each response was given a different score out of a 10-point ranging from 1.16 up to 4.34 as per its importance for each domain. The questions and the score given for each action was used from the research done in Ghana [14].

### Data quality control

The survey questionnaire is adapted from newborn services rapid health facility assessment tool [15], to evaluate the quality & access at primary health care level [16], service availability readiness assessment manual [17], rapid health facility assessment for core maternal, neonatal, and child health services at the primary level [18] and a health facility assessment tool for the quality of newborn care in rural Ghana [14]. Ten experienced health professionals in neonatal and child health services, at least first degree in nursing or health officers and who speak Amharic (the local language) were selected, trained and deployed in the field. Two days of training was provided for field workers and the questionnaire was pre-tested in two PHCUs which were not included in the study results.

### Operational definitions

#### Neonate/Newborn

An infant between 0 and 28 days old [19, 20].

#### Quality of service

The provision of services to the newborns as per the standard to improve desired health outcomes [21].

#### Primary health care unit

The lower level health care delivery functions in the primary health care that comprises five satellite health posts, one health centre and primary hospital [22].

#### Factors

Variables that cause, influence or determine to offer quality of service provision to newborns [19].

#### Low birth weight

If the weight of the newborn is between 1500 and 2500 g [23].

#### Very low birth weight

If the weight of the newborn is less than 1500 g [23].

#### Tracer essential medicines, equipment and supplies relevant for post-delivery newborn, and maternal &  newborn  care 

Seventeen and thirteen tracer functional essential equipment and supplies were selected for Primary hospitals, HCs, and HPs respectively to compute the mean percentage availability at each health facility. The following thirteen common essential equipment and supplies for all kinds of health facilities were selected, including newborn bag and masks (two sizes of neonatal masks), resuscitation table with health sources, infant/baby weighing scales, sink with soap or hand disinfectant for hand washing, towel for drying babies, cord ties, cord clamps, blood pressure machine (sphygmomanometer), fetoscopes, surgical gloves, graduated cup to measure expressed breastmilk, suction machine or nasal aspirators, and thermometers. In addition, four equipment including oxygen concentrators, cylinder, radiant warmer and vacuum extractors for delivery were included in the tracer equipment and supplies for PHs and HCs.

Tracer medicines for maternal and newborn health: the following ten tracer medicines were selected from Primary hospitals and Health centres including injectable oxytocics, ampicillin, gentamicin (20 mg/2 ml or 80 mg/2 m), anticonvulsant (magnesium sulphate or diazepam), dexamethasone, and vitamin K (phytomethadione), intravenous (IV) fluids with infusion sets, amoxicillin (dispersible tablet or syrup), nevirapine, and small size syringe and needles. On the other hand, only three tracer essential medicines including amoxicillin, gentamicin injection and small syringe and needles were selected at health posts level to compute the mean percentage score.

### Variables definitions

#### Dependent variable

The quality of newborn care service provision outcome variable was constructed from the five variables index, including the essential newborn care, care provided for low-birth-weight babies, monitoring postnatal care, signal functions for emergency newborn care, and newborn death audit. All five variables index included in the quality of newborn care (QNC) service provision are not on the same scale, and it was given equal weights and recalibrated to the range between zero and 10, with the highest score indicating better QNC service at a different level of health facilities.

The effectiveness of the neonatal health care services in the primary health care units’ composite index score was defined and measured by a composite index of quality of newborn care service provision, quality of sick young infants’ case management, and service utilization of sick young infants in the health facilities. All variables were given equal weights and recalibrated into a range between zero and 10 [24] with a higher score showing a better effectiveness of neonatal health care services. Cronbach’s alphas were also calculated to assess the internal reliability of the 11-health facility readiness items in measuring the underlying construct of the effectiveness of the neonatal health care services. It is expressed as a number between 0 and 1; and internal consistency The Cronbach’s alpha for the 11 facility readiness items was 0.5 which less than 0.7 from the acceptable value of alpha value [25].

#### Independent variables

Total number of skilled birth attendants available in the health facilities, percent of health workers received newborn health training in the last 1 year, percent availability of basic amenities in the health facilities, percent of essential equipment available in health facilities, percent of essential drugs available in health facilities, number of laboratory tests available in the health facilities, essential newborn care clinical scenario score, quality of care for very low birth weight babies’ clinical scenario score, overall newborn care knowledge of health providers managing sick children and newborns, percent of referral communication and percent of health facilities received supportive supervision in the last 3 months.

### Ethics approval and consent to participate

This study was conducted under the ethical principles of the Declaration of Helsinki. The study was approved by the Research and Ethics Committee from the Department of Health Studies, University of South Africa (UNISA) (Ref no: HSHDC/489/2015). The Amhara regional health bureau and West Gojjam Zone provided permission to conduct the study. Furthermore, the directors of PHs and HCs, and heads of the HPs also offered permission to conduct the study. Written informed consent was obtained from the study participants before the interview. Information on the study’s purpose, procedures, risks, burdens and benefits, as well as confidentiality and voluntariness of participation was provided to all potential participants as part of the informed consent process. Privacy and confidentiality of information provided by each participant were maintained throughout the study, and which were used exclusively for statistical purposes, were assured at all times. In addition, names and other specific addresses were not recorded and reported.

### Data analysis

Data was entered into the EpiData 3.1, exported to the Statistical Package for Social Science (SPSS Windows version 23) and STATA version 15 for analysis. Descriptive and regression multivariate analysis were applied to have a comprehensive description of the quantitative data and statistical relationship or association of variables. The analysis was presented in a table format with numbers, percentage, frequencies, means, *p*-value and confidence interval. A *p*-value of less than 0.05, was considered as major test value. In most of the variables, a mean was used to report the findings with a 95% confidence interval (CI).

## Results

### Socio-demographic characteristics

Out of the total 142 surveyed health facilities in the West Gojjam zone, 63 (44.4%) were rural health centres and 63 (44.4%) health posts (HPs) (Table 1).

**Table 1:** Distribution of surveyed facilities in the West Gojjam Zone

Health facility type	Frequency (***N*** = 142)	Percent
Primary hospitals (PHs)	3	2
Urban health centres (UHCs)	13	9.2
Rural health centres (RHCs)	63	44.4
Health posts (HPs)	63	44.4
**Total health facilities**	**142**	

Fifty-six (40.6%) of interviewed health providers in the maternity units had midwife diplomas or degrees, while HEWs accounted for 63 (45.7%). Similarly, of the total health providers interviewed in under-five clinics, 63 (44.4%) were HEWs, followed by nurses 51 (35.9%) (Table 2).

**Table 2:** Profile of interviewed health providers in the maternity units and under-five clinics in 142 PHs and HCs, and HPs by type of qualification and percent distribution

Background characteristics	Maternity units		Under-five clinics	
	Frequency (***N*** = 138)	Percent	Frequency (***N*** = 142)	Percent
Nurse all types (degree & diploma)	12	8.7	51	35.9
Midwife all types (degree & diploma)	56	40.6	5	3.5
Health Officer	7	5	22	15.5
HEWs	63	45.7	63	44.4
Medical doctor			1	0.7

### Emergency newborn care (EmNeC)

Emergency newborn care (EmNeC) was assessed by asking the seven signal functions including neonatal resuscitation with a bag and mask, kangaroo mother care (KMC) for premature or very low birth weight, injectable antibiotics for neonatal sepsis, corticosteroids in preterm labour, intravenous fluids for newborns, and bag and mask newborn resuscitation.

The highest score was for newborn resuscitation with bag and mask with 56 (71.8%) for primary hospitals and health centres and followed by injectable antibiotics for newborn sepsis with 85 (61.6%) of the health facilities including the HPs. However, corticosteroid for preterm labour was only practiced in 5 (6.5%) HCs and PHs. It is also the lowest score in all signal functions. Likewise, only 6 (7.7%) of HCs and PHs were administered intravenous fluids for the newborn and 41 (30%) of health facilities, including HPs were teaching mothers to express breast milk and feed with a small cup/spoon if the newborn is unable to feed. As per the report of 11 (18.3%) HPs, injectable antibiotics for the management of newborn sepsis were not practiced because of the lack of cases.

**Fig. 2:**
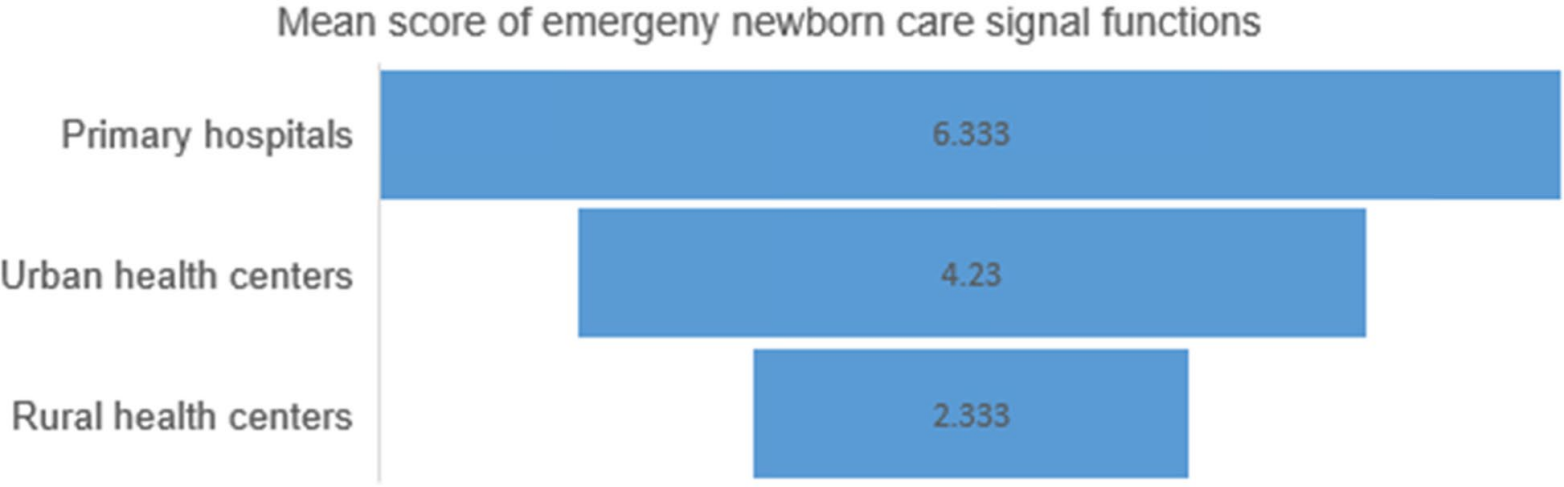
Provision of emergency newborn care signal functions in the last 6 months

The seven signal functions used to compute the mean provision of EmNEC signal functions in the last 6 months. Primary hospitals had a higher mean score, 6.3 [95%CI: 6.007–7.325] and Rural health centres had the lowest mean score, 2.3 [95%CI: 2.043–2.623] in the provision of EmNeC signal functions in the last 6 months before the survey (Fig. 2).

### Essential immediate newborn care practices

Health workers from primary hospitals and health centres were asked about the experience of essential newborn care practices, including breastfeeding, applying antibiotics in the eye, bathing, cord care, and weighing the baby. The application of chlorohexidine in the umbilical cord and putting the baby on the breast within 1 h of delivery were reported by 28 (35.4%) and 34 (43%) of health facilities respectively (Table 3).

**Table 3:** Availability of the immediate essential newborn care services in health facilities

Variable	Frequency	Percent
**The baby put in the mother abdomen once the baby is delivered**		
Primary hospitals (*N* = 3)	3	100
Urban health centres (*N* = 13)	13	100
Rural health centres (*N* = 63)	63	100
All health facilities (*N* = 79)	79	100
**First put the baby in the breast immediately after delivery**		
Primary hospitals (*N* = 3)	1	33.3
Urban health centres (*N* = 13)	7	53.8
Rural health centres (*N* = 63)	35	55.6
All health facilities (*N* = 79)	43	54.4
**First put the baby on the breast within one hour of delivery**		
Primary hospitals (*N* = 3)	2	66.7
Urban health centres (*N* = 13)	6	46.2
Rural health centres (*N* = 63)	26	41.3
All health facilities (*N* = 79)	34	43
**Apply antibiotics in the infant’s eye**		
Primary hospitals (*N* = 3)	3	100
Urban health centres (*N* = 13)	13	100
Rural health centres (*N* = 63)	63	100
All health facilities (*N* = 79)	79	100
**Bath after 24 h**		
Primary hospitals (*N* = 3)	3	100
Urban health centres (*N* = 13)	13	100
Rural health centres (*N* = 63)	57	90.4
All health facilities (*N* = 79)	73	92.4
**Nothing applied in the cord/dry cord care**		
Primary hospitals (*N* = 3)	1	33
Urban health centres (*N* = 13)	9	69
Rural health centres (*N* = 63)	37	58.7
All health facilities (*N* = 79)	47	59.5
**Apply chlorhexidine (CHX jell) the umbilical cord of the baby**		
Primary hospitals (*N* = 3)	2	66.7
Urban health centres (*N* = 13)	4	30.7
Rural health centres (*N* = 63)	22	34.9
All health facilities (*N* = 79)	28	35.4
**The weight of the baby is always monitored**		
Primary hospitals (*N* = 3)	3	100
Urban health centres (*N* = 13)	12	92.3
Rural health centres (*N* = 63)	61	96.8
All health facilities *N* = 79)	76	96.2

However, the rest of the essential and immediate newborn care services availability, including putting the baby on the abdomen of the mother once the baby is delivered, delaying bathing the baby for thermal protection, applying antibiotics in the eyes, and taking the weight of the baby was reported to be more than 90%. In addition, 4 (6.3%) of rural health centres were also applying Gentian violet to the umbilical cord.

### Care for very small/low birth weight babies

Among the six-lifesaving care for very small or low birth weight babies such as observation of babies for at least 1 day, kept the babies in the health facilities longer than usual, placed the babies in the incubator or radiant heater, kept the baby in the kangaroo mother care (KMC), and delayed first bath for the least 24 h, 110 (79.1%) of health facilities, including HPs delayed the first bath for the baby for at least 24 h and followed keeping the baby in KMC 102 (74.5%). The least performed care among the respondents from PHs and HCs was placing the baby in the radiant heater or warmer at 35 (45.5%).

The percentage mean score for the care of very small or low-birth-weight babies was also analysed against the level of care from the six actions for PHs and HCs from the six expected actions; and only three recommended actions including observation of babies for at least 1 day, kept the baby in the KMC and delayed the first bath for the least 24 h were used to compute the percentage mean score for the care of low-birth-weight babies in the HPs.

**Fig. 3:**
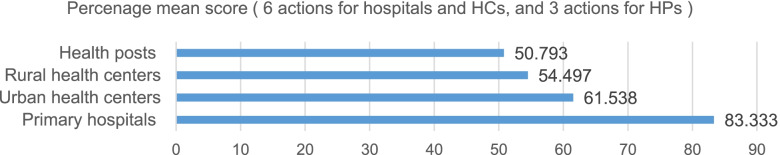
Percentage mean score for carrying of low birth weights babies

Figure 3 shows that, 83.3 [95%CI: 50.384–116.28] of the PHs practiced the recommend actions for the care of low-birth-weight babies; on the contrary, only half of the HPs practiced actions for the care of low-birth-weight babies (50.8 [95%CI: 43.510–58.076].

### Quality of essential newborn care: actions for resuscitation, follow-up and thermal care for newborns

**Fig. 4:**
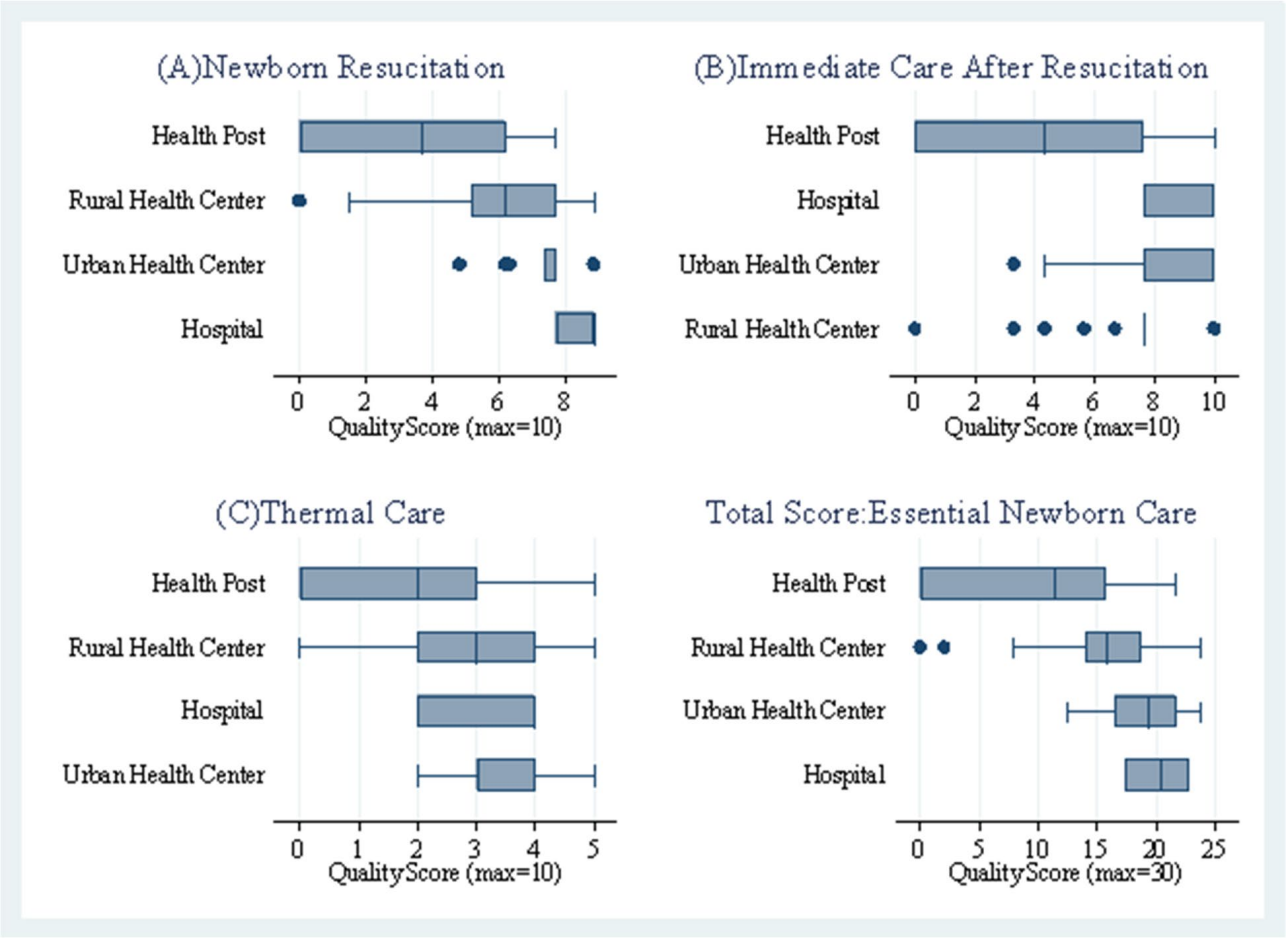
Essential newborn care scores by type of health facility. The lines in the box plots show the range of score, while the box captures the range of the middle 50%

As shown in Fig. 4A, in the first domain of live-saving newborn resuscitation, HPs had a mean score below 4 and Rural health centres scored slightly higher than 6. On the other hand, Primary hospitals had achieved a higher score, above 8, followed by Urban health centres with a score of less than 8. Also, for immediate actions after resuscitation of the newborn, HPs had the lowest mean score, slightly higher than 4 (Fig. 4B). All levels of health facilities achieved the lowest score than the rest of the two domains, with the mean score of less than 4 (Fig. 4C). Considering the five and three key resuscitation actions and follow-up care to save the lives of the newborns, and the five key actions to improve the quality of thermal care of the newborns, the mean score from the total 30 was computed for each health facility types. In line with this, among the four types of level of care, Primary hospitals had the highest mean score, 20.2 [95%CI: 17.076–23.377] and HPs had the lowest mean score, 9.9 [95%CI: 8.164–11.629]. Like Primary hospitals, 18.7 [95%CI: 16.783–20.658] and 15.9 [95%CI: 17.076–23.377] had the mean score for Urban health centres and Rural health centres respectively. It indicates that the mean score for quality of newborn care in the domains of newborn resuscitation, follow-up care after resuscitation and thermal care for newborns ranged below 10 for HPs and about 20 for PHs (Fig. 4 - Total score).

Among the total score of essential newborn care, PHs had the highest mean score, 20.2 [95%CI: 17.076–23.377] and HPs had the lowest mean score, 9.9 [95%CI: 8.164–11.629] (Fig. 4 - Total score).

### Availability of essential equipment for post-delivery newborn care

Based on the assessment done to know the availability of basic newborn care equipment and supplies in the health facilities, bag & masks and nasal suction and/or aspirators were available in 96 (70.6%) of health facilities including HPs. Out of the 58 HPs, only 20 (34.5%) possessed the newborn bag and mask. However, oxygen concentrators/cylinders were only available in 13 (16.5%) PHs and HCs; and only 6 (7.6%) health facilities had functional oxygen concentrators/cylinders. In line with this, only 5 (7.9%) of the Rural health centres had oxygen concentrators/cylinders, and only 1 (1.6%) had functional equipment.

Regarding keeping the thermal care of the newborn immediately after delivery, the availability of towels for drying babies and hats or caps for head coverings was only available in 3.7 and 1.5% of health facilities including health posts. The majority of assessed health facilities didn’t possess the supplies for keeping the thermal care of the baby. Despite all Primary hospitals and Urban health centres having radiant warmers in their respective health facilities, radiant warmers were available in only 28 (44.4%) of Rural health centres and 23 (36.5%) had functional equipment to provide the intended service. Similarly, an incubator was available in two out of the three assessed Primary hospitals.

Likewise, functional thermometer, baby scale and blood pressure machine (sphygmomanometer) were available in 129 (94.9%), 126 (92.6%) and 111 (81.6%) of Primary hospitals, Urban health centres, Rural health centres and HPs respectively. Nevertheless, cup to measure breast milk to care for very low birth weight and feeding problems for newborns was only available in 14 (10.3%) of health facilities including health posts.

**Fig. 5:**
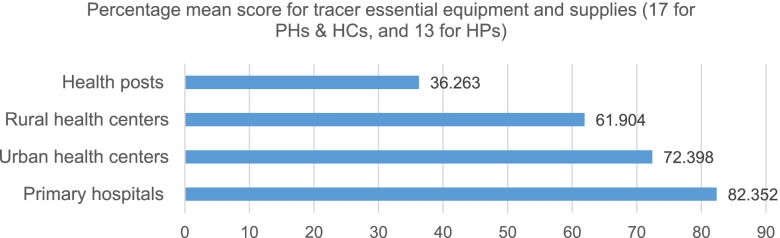
Percentage mean score for tracer essential equipment and supplies in health facilities

As shown in the Fig. 5, the highest, 82.3 [95%CI: 80.076–93.256] mean percentage score of functional essential tracer equipment and supplies were available in PHs; on the other hand, the lowest mean percentage score was documented for HPs at 36.3 [95%CI: 32.099–40.427].

### Availability of essential medicines for newborn and maternal health linked to newborn survival

For the management of neonatal sepsis, at least one valid doses of injectable ampicillin and injectable gentamicin (80 mg/2 ml) were available in 73 (93.6%) of health facilities excluding health posts. For the same purpose, injectable gentamicin (20 mg/2 ml), oral amoxicillin dispersible tablet and syrup were available in 39 (28.5%), 100 (73%) and 91 (65.55) Primary hospitals, Urban health centres, Rural health centres and HPs respectively. The injectable diazepam for the management of convulsions and injectable dexamethasone/corticosteroids to prevent breathing problems for newborns during preterm deliveries were available in 55 (69.6%) and 33 (41.8%) of health facilities excluding HPs.

Thus, a mean percentage score of 86.7 ([95%CI: 80.076–93.256], and 81.5 [95%CI: 78.497–84.579] of essential tracer medicines were available at Primary hospitals and Urban health centres respectively. On the contrary, the Rural health centres were less equipped with essential medicines with a mean percentage score of 74.8 [95%CI: 70.137–79.386]. While a 70-percentage mean of HPs [95%CI: 61.661–79.079] were equipped with the tracer essential medicines.

### Quality of newborn care service provision

**Fig. 6:**
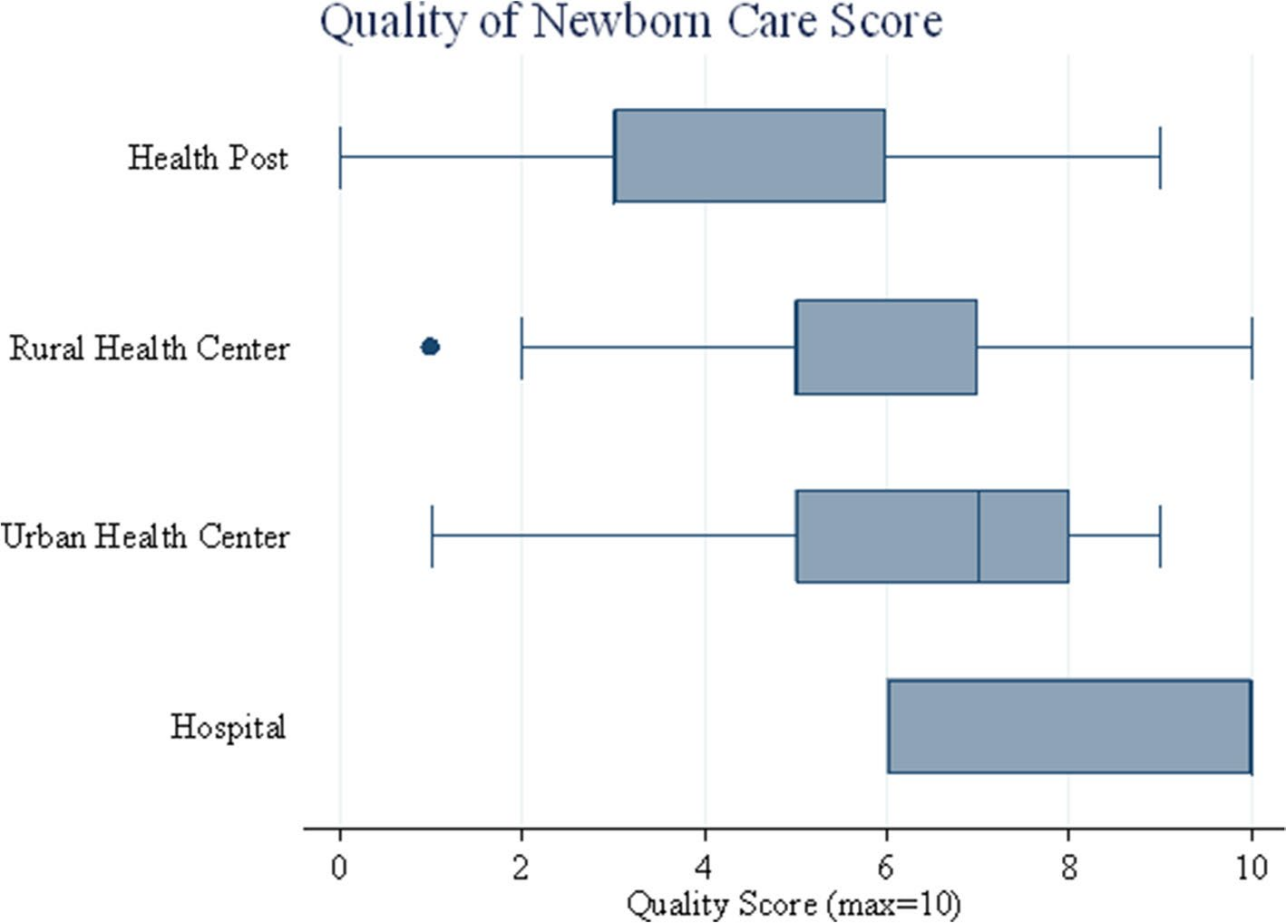
Quality of Newborn Care Score. The lines in the box plots show the range of scores, while the box captures the range of the middle 50%

Based on the five variables index included in the quality of newborn care (QNC) service provision, 8.7 [95%CI: 6.03–11.303], the highest mean QNC score was achieved by Primary hospitals followed by Urban health centres with a 6.4 mean [95%CI:5.168–7.601]. However, nearly half of the Rural health centres were providing QNC (5.7 [95%CI: 5.152–6.18], and below half of QNC was provided by HPs (4.5 [95%CI: 3.867–5.116]. This shows that the QNC given was high at the higher-level health facilities and lower in the lower level of health facilities (Fig. 6).

As shown in Table 4, the multiple regression analysis shows that, from the below 11 listed and computed facility readiness indicators (independent variables), only availability of essential equipment is significantly associated with the QNC provision in the health facilities (*p* < 0.05).

**Table 4:** Association between facility readiness indicators and quality of newborn care provision

Independent variable	Coefficient	***p***-value	95% LCI	95% UCI
Total number of skilled birth attendants available in the health facilities	0.025	0.097	−0.005	0.055
Percent of health workers received newborn health training in the last one year	−0.003	0.659	−0.015	0.010
Percent availability of basic amenities in the health facilities	0.017	0.087	−0.003	0.036
Percent of essential equipment available in health facilities	0.037	0.047*	0.000	0.074
Percent of essential drugs available in health facilities	−0.007	0.571	−0.033	0.018
Number of Laboratory tests available in the health facilities	0.272	0.124	−0.077	0.620
Essential newborn care clinical scenario score	0.100	0.062	−0.005	0.205
Quality of care for very low birth weight babies’ clinical scenario score	0.055	0.369	−0.067	0.177
Overall newborn care knowledge of health providers managing sick children and newborns (with score range zero to 10)	0.122	0.422	−0.179	0.423
Percent of referral communication	0.014	0.195	−0.007	0.035
Percent of health facilities received supportive supervision in the last 3 months	0.132	0.762	−0.736	1.000
cons	−2.646	0.111	−5.916	0.624

^*^P value < 0.05

### Effectiveness of the neonatal healthcare services

**Fig. 7:**
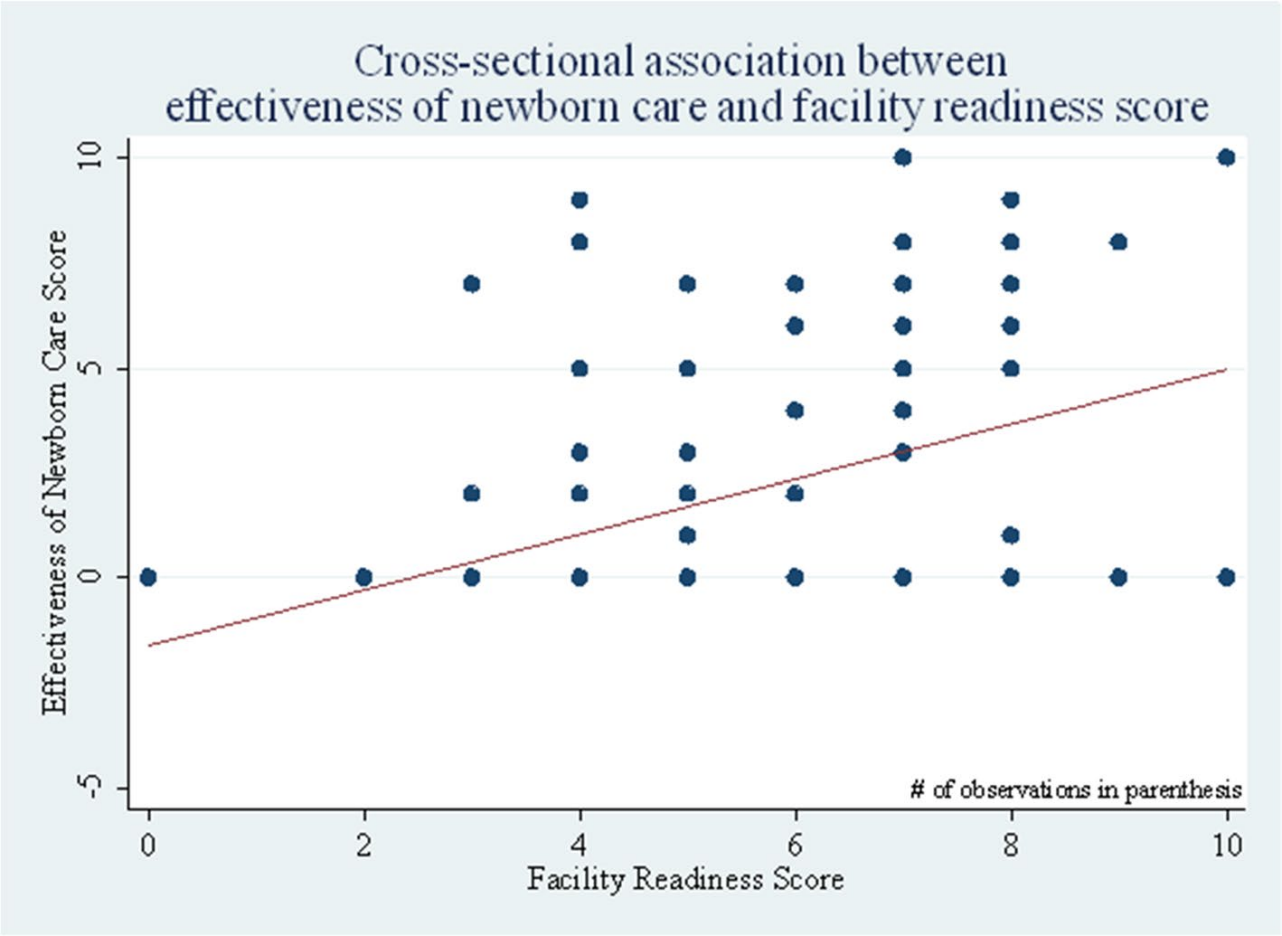
The cross-sectional association between effectiveness of newborn care and facility readiness score

Overall, the effectiveness of the newborn care services in the primary health care ranged between zero and ten, for every unit increase of the facility readiness score, there was a corresponding average of 0.45 percentage points [95%CI: 0.134–0.768] increase in the effectiveness of the newborn care services. This implies that the effectiveness of the neonatal health care services has a statistically significant association with the health facilitates readiness score (*p* < 0.05) (Fig. 7).

## Discussion

This study has shown that the quality of neonatal healthcare provision is low to moderate in the primary healthcare units. Especially, Health posts and Rural health centres offered a lower quality of essential newborn care. Likewise, the provision of the EmNeC signal functions to save the lives of the sick young infants in the rural health centres was critically low and was relatively better provided in the Primary Hospitals. Overall, the availability of essential equipment has an impact on the quality of neonatal care provision in the health facilities, and health facility readiness is contributing to the effectiveness of neonatal healthcare services provision.

Newborn health care delivery across the continuum of care in primary health care differs due to the readiness of health facilities with human resources, infrastructure, medicines and equipment, and others. As a result, the level of care for newborns differs from primary hospital to health centre and health posts. In this study, the comparison of health facilities was done based on what was expected at each level of care.

Regarding the performance of the seven EmNeC signal functions, in the study done in Ethiopia [26], among health facilities that were assessed in the Amhara region, 76% performed newborn resuscitation with bag and mask, and 51% performed KMC for small babies in the last 3 months prior to the survey. Antibiotics for neonatal sepsis and antibiotics for preterm premature rupture of membranes were performed in the 39 and 42% health facilities. Another study done in Ethiopia also revealed that neonatal resuscitation was available in 59% of health facilities in Amhara region, followed by KMC for premature/very small babies, antibiotics for preterm or prolonged premature rupture of membranes, and injectable antibiotics for neonatal sepsis were available in 45, 33 and 30% of health facilities in the Amhara region respectively [27]. In agreement with the existing evidence, this study showed that the highest score was for newborn resuscitation with a bag and mask with 71.8% and followed by injectable antibiotics for newborn sepsis with 61.6% of the health facilities. In addition, the corticosteroid for preterm labour was only performed in 6.5% of health facilities (HCs and PHs). It is also the lowest score in all EmNeC signal functions. Likewise, only 7.7% of health facilities administered intravenous fluids for newborns.

Overall, the likelihood of getting EmNeC signal functions to save the lives of the sick young infants in the Rural health centres in the West Gojjam Zone was critically low and were better provided in PHs. This is supported by the existing evidences, only 3% of health facilities in the Amhara region, Ethiopia were providing fully EmNeC signal functions, and there were no fully EmNeC facilities in rural areas and no health centres were provided fully EmNeC at national level [28] and the mean availability of EmNeC signal functions was 30% of health facilities in Amhara region [29]). This is also supported by the assessment done in health facility capacity to provide newborn care in the five countries [30], hospitals and facilities in urban areas were in a better position in both service availability and readiness; however, the readiness and availability of essential newborn care services in the rural areas facilities were reported as a substantial equity gap for people accessing lower-level health. Similarly, in the two studies done in India, the district hospitals were slightly better equipped to provide quality maternal and newborn care in terms of infrastructure, equipment and supplies by comparison to primary health centres [31] and the higher facilities were better equipped to provide delivery and newborn care among the public primary health facilities [32].

Research results by Vesel et al. on the quality of newborn care in rural Ghana [14] highlights that lower level facilities achieved only low-to-moderate scores for newborn resuscitation, immediate care after resuscitation and thermal care for the newborns and provided overall low quality of essential newborn care. Consistently with the evidence, this study shows that the mean score for quality of newborn care with the domains of 30 newborn resuscitation, follow-up care after resuscitation and thermal care for newborns actions ranged below 10 for Health posts, 15.9 for Rural health centres, 18.7 for Urban health centres and about 20 for Primary hospitals. This implies that the lower-level health facilities including Health posts and Rural health centres had a lower quality essential newborn care.

The service availability and readiness assessment in the Amhara region, Ethiopia showed that, neonatal bags and masks, and infant weighing scales were found in 46 and 90% of health facilities [29]. In consistent with the existing evidence, in this study, bag & masks and nasal suction and/or aspirators were available in 70.6% of health facilities including HPs. According to the United Nations (UN) commission, resuscitation devices for newborn asphyxia are among the 13 life-saving commodities [33].

Regarding keeping the thermal care of the newborn immediately after delivery, the availability of towels for drying babies and hats or caps for head covering were only available in 3.7 and 1.5% of health facilities including HPs; which is lower than the EmONC assessment done in 2016 in Ethiopia. In addition, 82.3 mean percentage score of functional essential equipment and supplies were available in Primary hospitals; followed by 72.4 in Urban health centres. Only 61.9 percentage score of tracer essential equipment and supplies were available in the Rural health centres. Overall, HPs and Rural health centres were facing a shortage of essential equipment and supplies to provide key intended services for mothers and newborns.

## Conclusion

In this study, the Rural health centres and Health posts provide sub-optimal or low-quality neonatal healthcare services. Thus, these facilities should be targeted to improve their readiness to provide the quality of newborn services as per their level of care. In addition, the availability of essential equipment and health facility readiness are significantly associated with the quality of neonatal care provision and the effectiveness of the neonatal healthcare services respectively. Therefore, the primary healthcare units should be assessed regularly for their readiness and fulfill the missing component to ensure the quality of newborn care services as per their level of care.

### Limitation of the study

Because of the resource limitation, to assess the quality of newborn care service provision, case observation was not done and the neonatal healthcare provision from the women and family perspective was not included as part of this study.

## Data Availability

Full data set and materials pertaining to this study can be obtained from the correspondent author on a reasonable request.

## Abbreviations

CI: Confidence Interval
EmNeC: Emergency Newborn Care
HCs: Health Centres
HEWs: Health Extension Workers
HWs: Health Workers
KMC: Kangaroo Mother Care
LBs: Live Births
PHCUs: Primary Health Care Units
PHs: Primary Hospitals
PNC: Postnatal Care
QNC: Quality of Newborn Care
RHCs: Rural Health Centres
UHCs: Urban Health Centres
